# Perioperative and oncological outcomes following minimally invasive versus open pancreaticoduodenectomy for pancreatic duct adenocarcinoma

**DOI:** 10.1007/s00464-020-07641-1

**Published:** 2020-07-06

**Authors:** Rui Sun, Jiawen Yu, Yifan Zhang, Zhika Liang, Xianlin Han

**Affiliations:** 1 Department of General Surgery, Peking Union Medical College Hospital, No.1 Shuaifuyuan Wangfujing Dongcheng District, Beijing, 100730 China; 2 Chinese Academy of Medical Sciences and Peking Union Medical College, Beijing, China; 3 Department of Pediatric Surgery, Capital Institute of Pediatric, Beijing, China; 4 Department of Agronomy and Horticulture, University of Nebraska-Lincoln, Lincoln, USA

**Keywords:** Pancreatic ductal carcinoma, Pancreaticoduodenectomy, Laparoscopic surgery, Robotic surgical procedures, Minimally invasive surgery, Meta-analysis

## Abstract

**Background:**

The outcomes of minimally invasive pancreaticoduodenectomy have not been adequately compared with those of open pancreaticoduodenectomy in patients with pancreatic ductal adenocarcinoma. We performed a meta‐analysis to compare the perioperative and oncological outcomes of these two pancreaticoduodenectomy procedures specifically in patients with pancreatic ductal adenocarcinoma.

**Methods:**

Before this study was initiated, a specific protocol was designed and has been registered in PROSEPRO (ID: CRD42020149438). Using the Preferred Reporting Items for Systematic Reviews and Meta-analyses (PRISMA) guidelines, PubMed, EMBASE, Web of Science, Cochrane Central Register, and ClinicalTrials.gov databases were systematically searched for studies published between January 1994 and October 2019. Overall survival, disease-free survival, and time to commencing adjuvant chemotherapy were the primary endpoint measurements, whereas perioperative and short-term outcomes were the secondary endpoints.

**Results:**

The final analysis included 9 retrospective cohorts comprising 11,242 patients (1377 who underwent minimally invasive pancreaticoduodenectomy and 9865 who underwent open pancreaticoduodenectomy). There were no significant differences in the patients’ overall survival, operative time, postoperative complications, 30-day mortality, rate of vein resection, number of harvested lymph nodes, or rate of positive lymph nodes between the two approaches. However, disease-free survival, time to starting adjuvant chemotherapy, length of hospital stay, and rate of negative margins in patients who underwent minimally invasive pancreaticoduodenectomy showed improvements relative to those in patients who underwent open surgery.

**Conclusions:**

Minimally invasive pancreaticoduodenectomy provides similar or even improved perioperative, short-term, and long-term oncological outcomes when compared with open pancreaticoduodenectomy for patients with pancreatic ductal adenocarcinoma.

**Electronic supplementary material:**

The online version of this article (10.1007/s00464-020-07641-1) contains supplementary material, which is available to authorized users.

Pancreatic ductal adenocarcinoma (PDAC) is an aggressive malignancy that is the 14th most common cancer and the seventh leading cause of cancer-related mortality worldwide [1]. Despite the application of various neoadjuvant and adjuvant treatment protocols, pancreaticoduodenectomy (PD) remains the only curative treatment for patients with adenocarcinoma of the pancreatic head. PD is one of the most complex procedures in gastroenterological surgery, and requires extensive visceral organ dissection and complex reconstructive digestive anastomoses; therefore, it is normally performed using an open approach.

The first laparoscopic PD (LPD) was reported by Gagner and Pomp in 1994 [2], while the first robotic PD (RPD) was described by Giulianotti et al. in 2003 [3]. Since then, minimally invasive PD (MIPD), which encompasses LPD and RPD, is increasingly used worldwide; however, it remains a challenging procedure because of the technical limitations of laparoscopy or robot control as well as the steep learning curve when training physicians. Therefore, the feasibility and safety of the minimally invasive approach remain controversial.

Recent studies [4, 5] and meta-analyses [6, 7] showed that the outcomes of MIPD were similar to or more favorable than open PD (OPD) with respect to the incidence of postoperative morbidity, short-term oncologic outcomes, and long-term overall survival rates. Furthermore, MIPD is associated with lower estimated intraoperative blood loss, shorter length of hospital stay (LOS), higher rate of R0 resection, and the harvesting of a greater number of lymph nodes. However, these studies involved patients with a variety of disease histologies; therefore, objective conclusions regarding the oncologic outcomes of patients with this malignancy, especially PDAC, were unclear. Other related meta-analyses [8, 9] involved relatively few patients with PDAC. According to our search, no meta-analysis was performed specifically to investigate the perioperative, short-term, and long-term oncological outcomes of patients who underwent MIPD for PDAC. In this study, we carefully screened and selected studies that specifically investigated patients with PDAC. The aim of our meta-analysis was to meaningfully assess the perioperative, short-term, and long-term oncologic outcomes of these patients, with the primary investigative endpoints being overall survival (OS), disease-free survival (DFS), and the time to starting postsurgical adjuvant chemotherapy.

## Materials and methods

Before this study was initiated, we designed a specific protocol which has been registered in PROSEPRO. The ID is CRD42020149438 (details of registration is included in Supplementary Materials). Thus, this study was performed in accordance with the recommendations of the Preferred Reporting Items for Systematic Reviews and Meta-analyses (PRISMA) guidelines [10]. This article does not contain any studies with human participants performed by any of the authors; therefore, there was no requirement for IRB approval.

### Data sources and search methods

We systematically searched the PubMed, EMBASE, Web of Science, Cochrane Central Register, and ClinicalTrials.gov databases for studies published in English between 1994 and October 2019. The search Medical Subject Heading (MeSH) terms were “laparoscopic pancreaticoduodenectomy” OR “robotic pancreaticoduodenectomy” AND “pancreatic ductal adenocarcinoma,” as well as all associated entry words retrieved using the MeSH index (details of our search strategy are included in Supplementary Materials). There was no language restriction. We also reviewed the introduction and discussion sections of the retrieved manuscripts, relevant review articles, and published meta-analyses to identify additional trials. Two authors (Rui Sun and Jiawen Yu) independently conducted the literature search, screened the abstracts, and selected the relevant trials.

### Inclusion criteria

Studies published between January 1994 and October 2019 were considered eligible if they met the following inclusion criteria: (1) the investigated population comprised patients with PDAC; (2) the interventions compared were LPD or RPD versus OPD; and (3) the study investigated at least one of the following outcomes: operative time, intraoperative blood transfusion, postoperative morbidity and mortality, LOS, rate of vein and R0 resection, number of lymph nodes retrieved, time to starting adjuvant treatment, DFS, and OS.

### Exclusion criteria

The exclusion criteria are as follows: (1) review articles; (2) meeting abstracts; (3) irrelevant studies such as those that investigated only a single surgical technique; (4) insufficient information available in the English abstract; (5) studies with no comparative data; and (6) studies without PDAC data. If papers had overlapping data, those describing the smaller-scale studies were excluded.

### Quality assessment

We adopted the Newcastle–Ottawa Scale (NOS) [11], which is designed specifically for observational investigations, to assess the quality of the selected studies. The NOS focuses on 3 separate sections of a case–control or cohort study, with the number of stars representing the assessment score. The maximum achievable score under the NOS is 9 stars, including 4 for the selection process, 2 for comparability, and 3 for exposure and outcome. A score of ≥ 6 stars is considered indicative of high quality. Two investigators independently assessed the selected studies.

### Data extraction

Two investigators independently extracted the following information: first author, year of publication, study type, mean age, population size, tumor size, and main outcomes; the latter included operative time, intraoperative blood transfusion, postoperative morbidity and mortality, LOS, rate of vein and R0 resection, number of lymph nodes retrieved, time to adjuvant treatment, DFS, and OS. The evaluators resolved any disputes via consensus during the screening processes.

### Statistical analysis

Statistical analyses were conducted using Review Manager 5.3 (Cochrane Collaboration). The relative risk (RR) and mean difference (MD) with the 95% confidence interval (CI) were used as the measures of dichotomous and continuous variables, respectively. Natural logarithm hazard ratios (HRs) and standard errors were used as summary statistics for DFS and OS data. Using the methods of Wan et al. [12] and Luo et al. [13], medians with ranges as well as the mid-quartile range were converted into means with standard deviations. For studies in which natural logarithm HRs and standard errors or corresponding 95% CIs were not available, estimates from the published survival curves were calculated using the method suggested by Tierney et al. [14]. *P*-values less than 0.05 indicated statistical significance. Heterogeneity was quantified by the *I*^2^ statistic; a study with an *I*^2^ less than 50% was considered to have no heterogeneity, and the fixed effects model was then applied to pool the results; otherwise, the random effects model was used.

## Results

### Search results and characteristics of the included studies

**Fig. 1:**
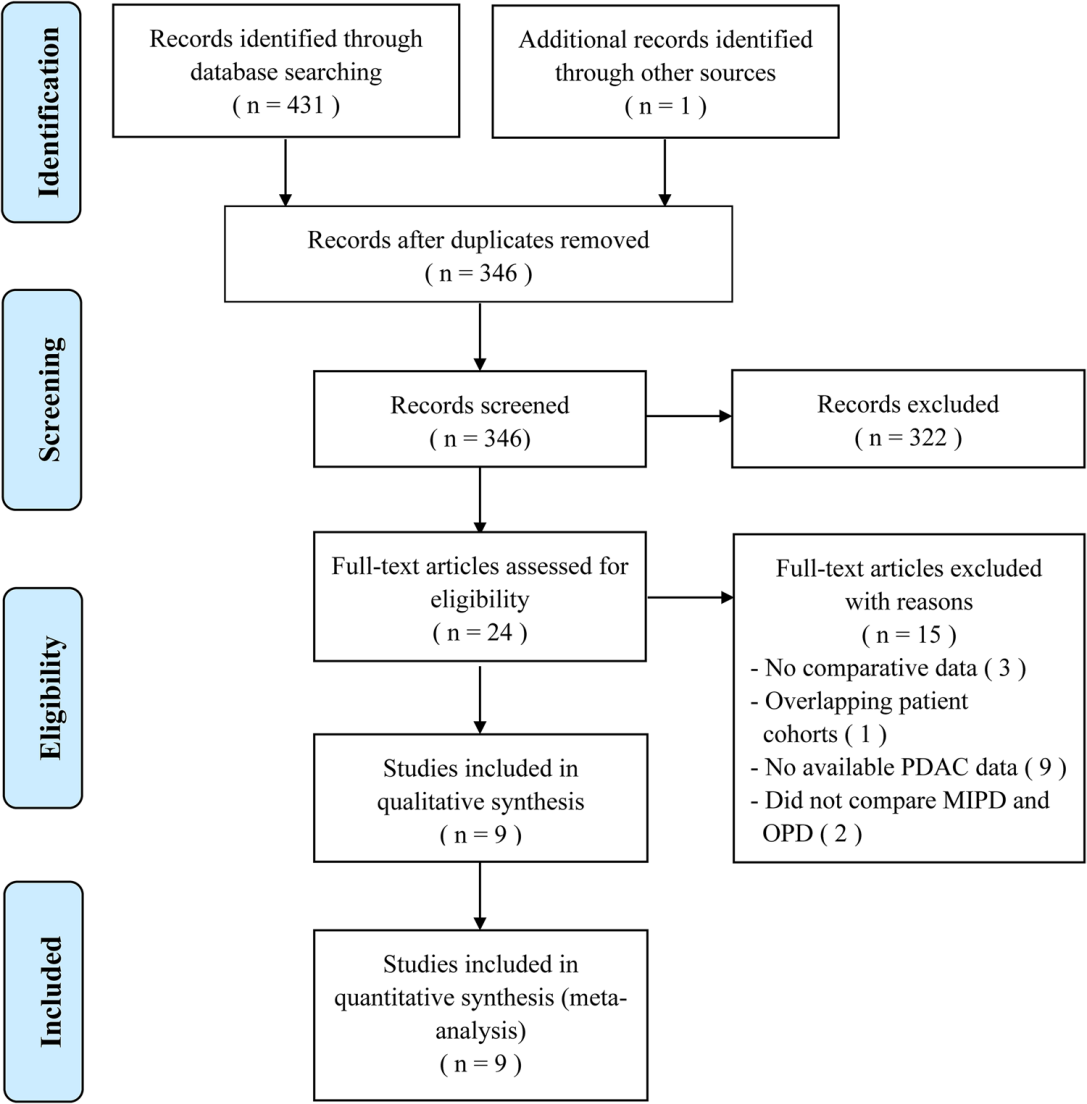
Flowchart showing the protocol of the meta-analysis. *MIPD* minimally invasive pancreaticoduodenectomy, *OPD* open pancreaticoduodenectomy, *PDAC* pancreatic duct adenocarcinoma

**Fig. 2:**
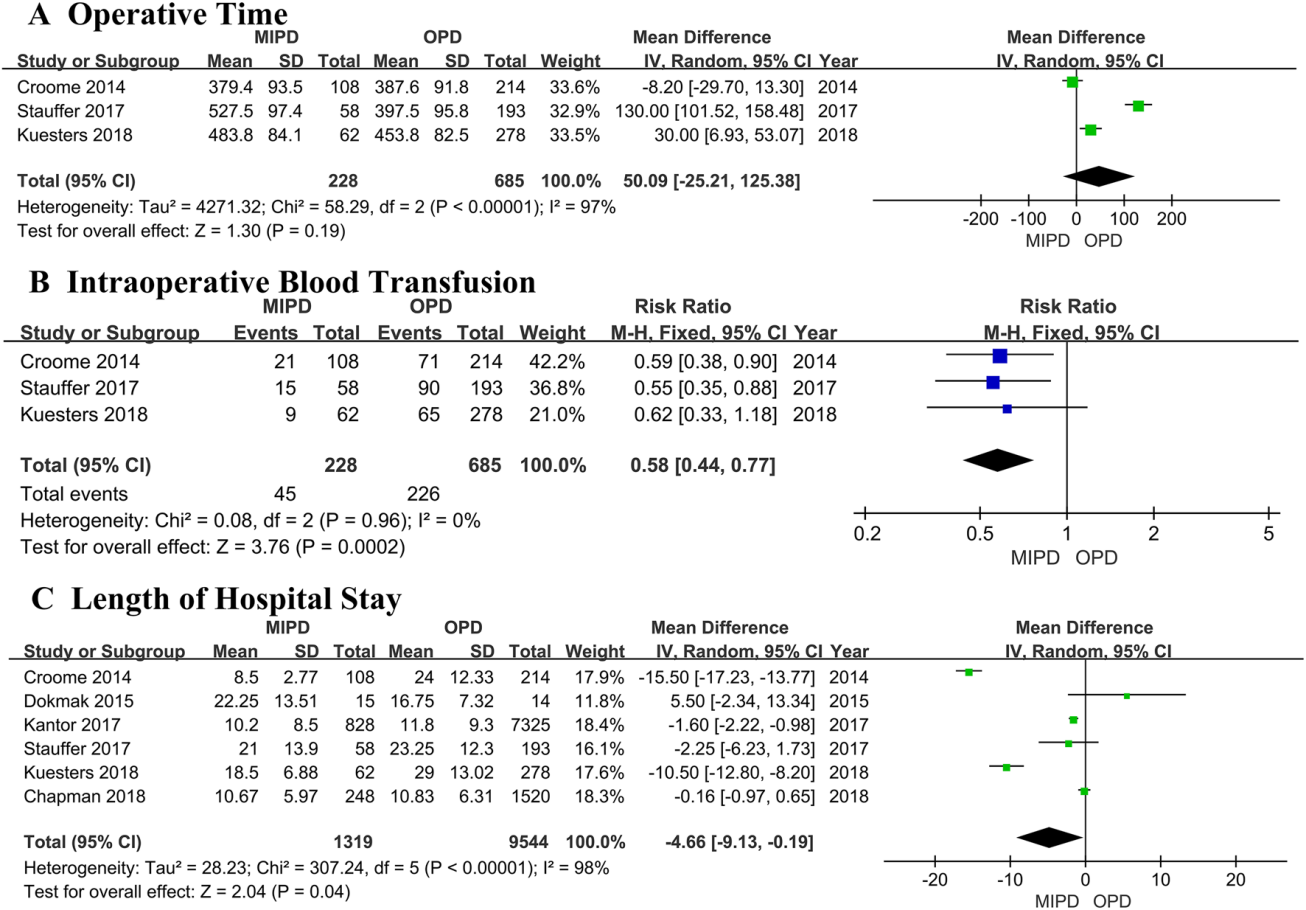
Comparison of perioperative outcomes between patients who underwent minimally invasive pancreaticoduodenectomy (MIPD) and those who underwent open pancreaticoduodenectomy (OPD) for pancreatic duct adenocarcinoma

**Fig. 3:**
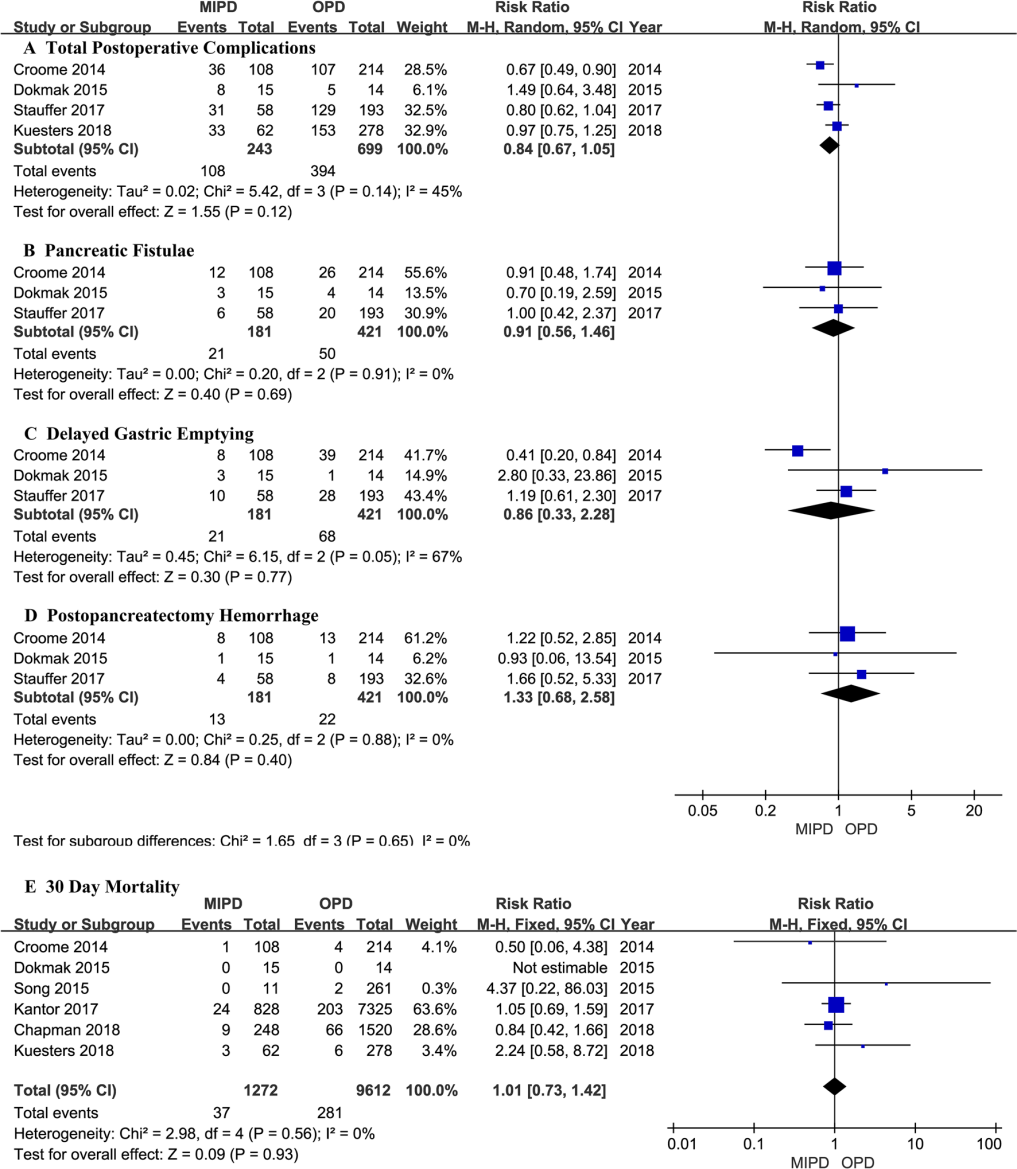
Comparison of postoperative and 30-day mortality between patients who underwent minimally invasive pancreaticoduodenectomy (MIPD) and those who underwent open pancreaticoduodenectomy (OPD) for pancreatic duct adenocarcinoma

**Fig. 4:**
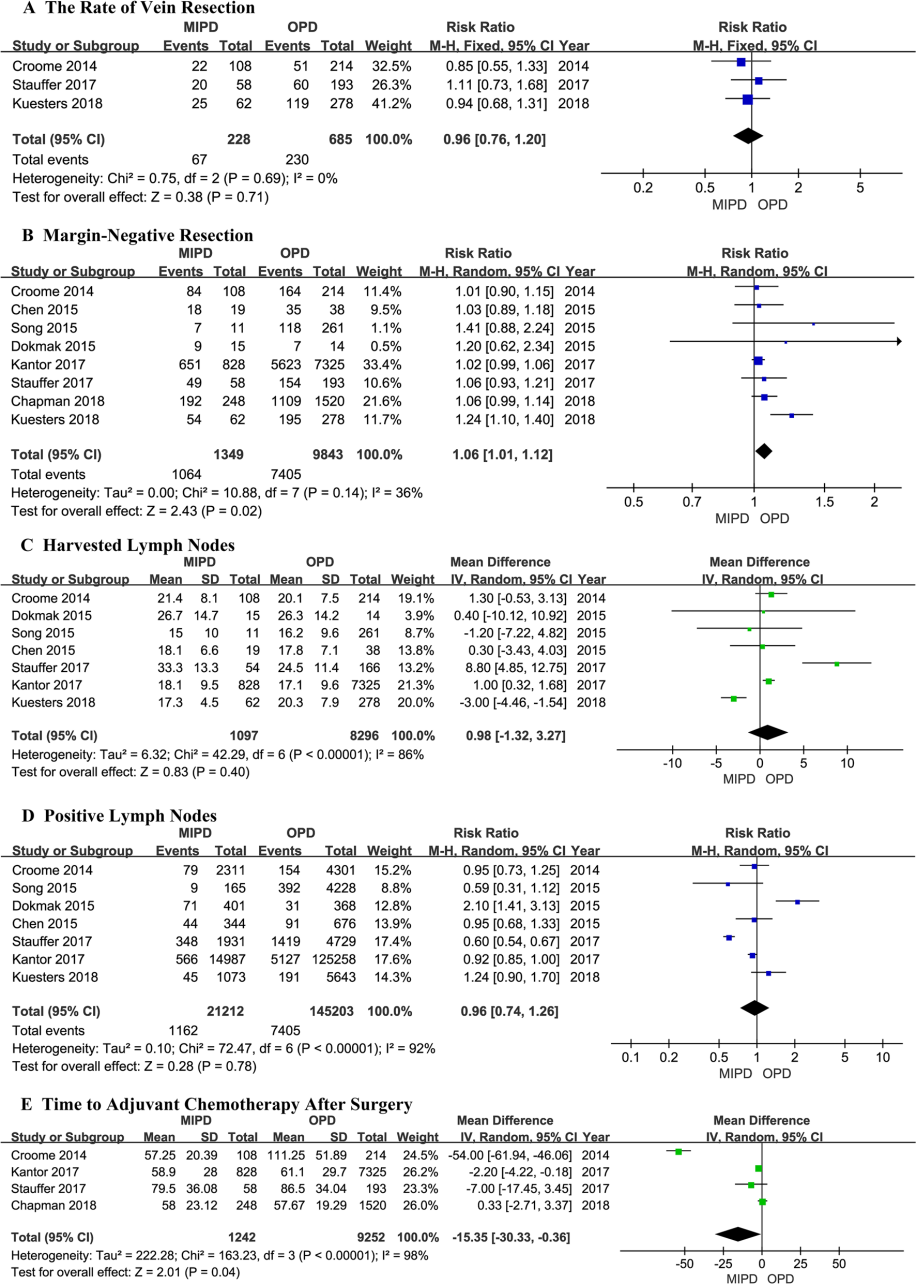
Short-term oncological outcomes of patients who underwent minimally invasive pancreaticoduodenectomy (MIPD) and those who underwent open pancreaticoduodenectomy (OPD) for pancreatic duct adenocarcinoma

**Fig. 5:**
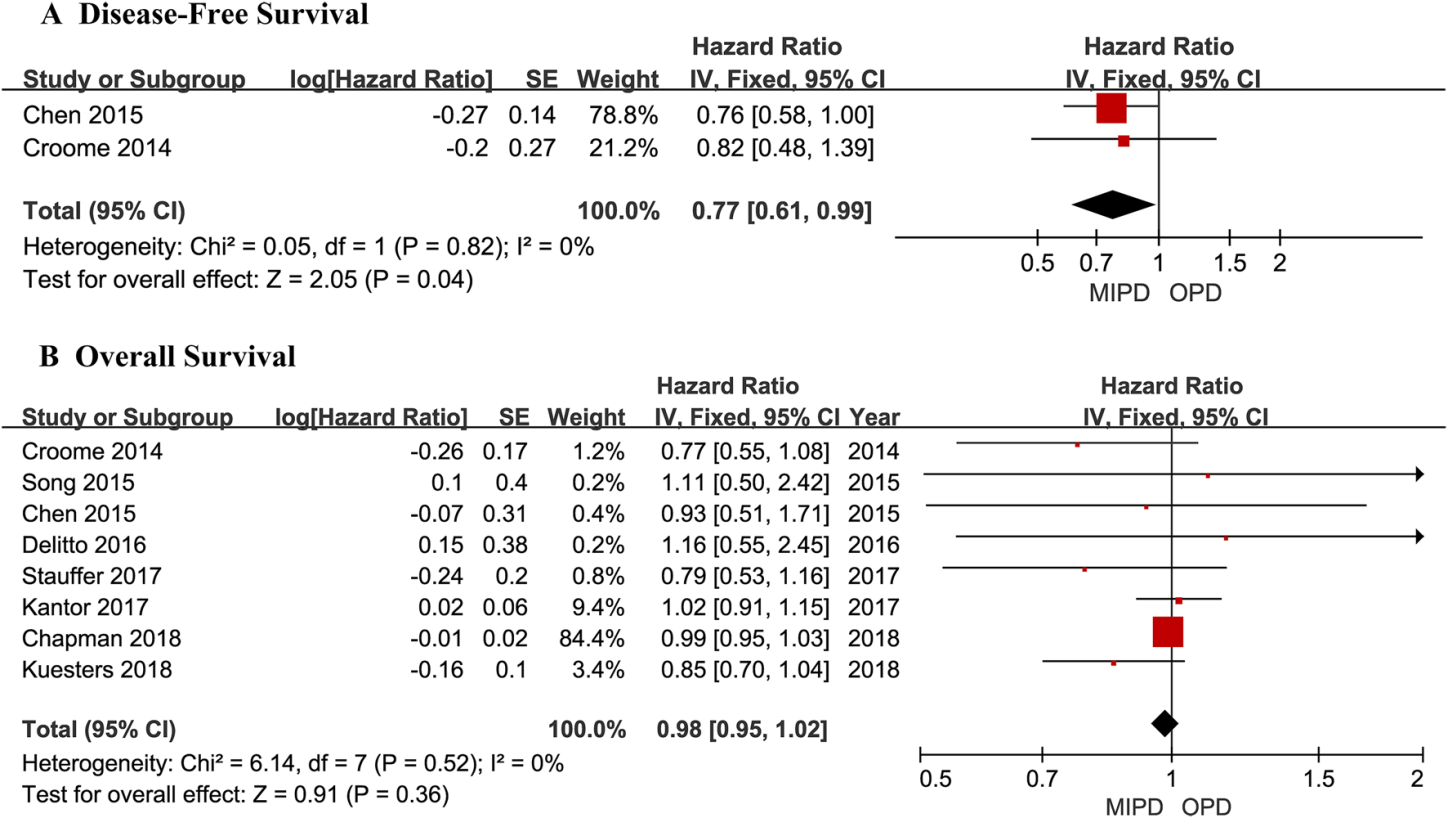
Long-term oncological outcomes of patients who underwent minimally invasive pancreaticoduodenectomy (MIPD) and those who underwent open pancreaticoduodenectomy (OPD) for pancreatic duct adenocarcinoma

A flowchart of our analysis protocol is shown in Fig. 1. Our analysis included 9 retrospective cohort studies [15–23] comparing MIPD and OPD in a total of 11,242 patients with PDAC (1377 and 9865 underwent MIPD and OPD, respectively). The characteristics and qualities of these 9 studies are listed in Table 1, while the main results are shown in Table 2. All the analysis results including operative time, intraoperative blood transfusion, LOS, postoperative complications and mortality, and short- and long-term oncological outcomes are shown in Figs. 2, 3, 4, and 5. Among the 9 studies, 8 [15, 17–23] compared LPD and OPD and only 1 [16] compared RPD and OPD. The means of the patients’ ages in each study ranged from 65.4 to 79.6 years with a majority in both the MIPD and OPD cohorts being of comparable age; only patients of both groups in Chapman et al.’s study (79.6 versus 79.6 years; *P* = 0.99) [22] were older than those in other studies. There were no significant differences between the proportions of male and female patients with PDCA.

**Table 1:** Characteristics and qualities of the 9 studies included in the meta-analysis

Authors, year	Study type	Surgery type	Samples		Sex (M/F)		Age (mean ± SD year)		Tumor size (mean ± SD cm)		Follow-up (months)		NOS
			MIPD	OPD	MIPD	OPD	MIPD	OPD					
									MIPD	OPD	MIPD	OPD	
Croome, 2014 [15]	Retrospective	LPD/OPD	108	214	51/53	131/83	66.6 ± 9.6	65.4 ± 10.9	3.3 ± 1.0	3.3 ± 1.3	16.5	15.1	8
Chen, 2015 [16]	Retrospective	RPD/OPD	19	38	Not clear		Not clear		3.0 ± 0.9	3.1 ± 1.0	22 ± 10	21 ± 8	7
Dokmak, 2015 [17]	Retrospective	LPD/OPD	11	261	Not clear		Not clear		2.6 ± 0.72	3.0 ± 0.44	Not clear		6
Song, 2015 [18]	Retrospective	LPD/OPD	15	14	Not clear		68.1 ± 7	61.8 ± 10.5	2.8 ± 0.6	3.0 ± 1.2	Not clear		6
Delitto, 2016 [19]	Retrospective	LPD/OPD	28	22	Not clear		Not clear		Not clear		Not clear		7
Kantor, 2017 [20]	Retrospective	LPD/OPD	828	7325	Not clear		65.9 ± 10.7	65.7 ± 10.4	Not clear		18		8
Stauffer, 2017 [21]	Retrospective	LPD/OPD	58	193	32/35	96/97	66.3 ± 9.5	64.5 ± 9.8	3.8 ± 2.1	5.4 ± 2.5	19.6 ± 17.4	24.5 ± 27.4	8
Chapman, 2018 [22]	Retrospective	LPD/OPD	248	1520	132/116	721/799	79.6	79.5	Not clear		17.1	14	8
Kuesters, 2018 [23]	Retrospective	LPD/OPD	62	278	31/31	137/141	71	68	3.3 ± 1.59	4.7 ± 2.2	Not clear		7

*LPD* laparoscopic pancreaticoduodenectomy, *RPD* robotic pancreaticoduodenectomy, *OPD* open pancreaticoduodenectomy, *MIDP* minimal invasive pancreaticoduodenectomy, *M/F* male/female, *NOS* Newcastle–Ottawa Scale, *SD* standard deviation

**Table 2:** Main outcomes of patients in the nine studies included in this meta-analysis

Study	Samples	OT (mins) Mean ± SD	BT	LOS (days) Mean ± SD	POPF	DGE	PH	TC	30D Mor	No. lymph nodes resected	Nodes (+)	VR	R0	ACT	DSF HR (M/O) (95%CI)	OS HR (M/O) (95%CI)
Croome [15]	MIPD:108	379.4 ± 93.5	21	8.5 ± 2.77	12	8	8	36	1	21.4 ± 8.1	79	22	84	57.25 ± 20.39	0.76 (0.58, 1.00)	0.77 (0.55, 1.08)
	OPD: 214	387.6 ± 91.8	71	24 ± 12.33	26	39	13	107	4	20.1 ± 7.5	154	51	164	111.25 ± 51.89		
Chen [16]	MIPD:19	NC	NC	NC	NC	NC	NC	NC	NC	18.1 ± 6.6	44	NC	18 35	NC	0.82 (0.48, 1.39)	0.93 (0.51, 1.71)
	OPD: 38									17.8 ± 7.1	91					
Dokmak [17]	MIPD:15	NC	NC	22.25 ± 13.51	3	3	1	8	0	26.7 ± 14.7	71	NC	9	NC	NC	NC
	OPD: 14			16.75 ± 7.32	4	1	1	5	0	26.3 ± 14.2	31		7			
Song [18]	MIPD:11	NC	NC	NC	NC	NC	NC	NC	0	15 ± 10	9	NC	7	NC	NC	1.11 (0.50, 2.42)
	OPD:261								2	16.2 ± 9.6	392		118			
Delitto [19]	MIPD:28	NC	NC	NC	NC	NC	NC	NC	NC	NC	NC	NC	NC	NC	NC	1.16 (0.55, 2.45)
	OPD: 22															
Kantor [20]	MIPD:828	NC	NC	10.2 ± 8.5	NC	NC	NC	NC	24	18.1 ± 9.5	566	NC	651	58.9 ± 28.0	NC	1.02 (0.91, 1.15)
	OPD:7325			11.8 ± 9.3					203	17.1 ± 9.6	5127		5623	61.1 ± 29.7		
Stauffer [21]	MIPD:58	527.5 ± 97.4	15	21 ± 13.9	6	10	4	31	NC	33.3 ± 13.3	348	20	49	79.5 ± 36.08	NC	0.79 (0.53, 1.16)
	OPD:193	397.5 ± 95.8	90	23.25 ± 12.3	20	28	8	129		24.5 ± 11.4	1419	60	154	86.5 ± 34.04		
Chapman [22]	MIPD:248	NC	NC	10.67 ± 5.97	NC	NC	NC	NC	9	NC	160	NC	192	58 ± 23.12	NC	0.99 (0.95, 1.03)
	OPD:1520			10.83 ± 6.31					66		988		1109	57.67 ± 19.29		
Kuesters [23]	MIPD:62	483.8 ± 84.1	9	18.5 ± 6.88	NC	NC	NC	25	3	17.3 ± 4.5	45	25	54	NC	NC	0.85 (0.70, 1.04)
	OPD:278	453.8 ± 82.5	65	29 ± 13.02				107	6	20.3 ± 7.9	191	119	195			

*MIPD* minimal invasive pancreaticoduodenectomy, *OPD* open pancreaticoduodenectomy, *OT* operative time, *BT* intraoperative blood transfusion, *LOS* length of hospital stay, *POPF* postoperative pancreatic fistulae, *DGE* delayed gastric emptying, *PH* postpancreatectomy hemorrhage, *TC* total postoperative complications, *30D Mor* 30-day mortality, *No.* number, *VR* vein resection, *R0* margin-negative resection, *ACT* time to adjuvant chemotherapy after surgery, *DSF* disease-free survival, *HR* hazard ratio, *M/O* open pancreaticoduodenectomy as reference, *OS* overall survival, *CI* confidence interval

### Perioperative outcomes

#### Operative time

Three studies [15, 21, 23] that encompassed 913 patients investigated operative times (including 228 and 685 patients who underwent MIPD and OPD, respectively). The operative time was longer for the MIPD group in 2 of these studies, and was longer for the OPD group in the third. The pooled estimates of these studies showed that the duration of surgery was not significantly different between the MIPD and OPD groups (MD 50.09; 95% CI − 25.21 to 125.38; *P* = 0.11). The analysis found statistically significant heterogeneity (*I*^2^ = 97%); thus, a random effects model was adopted (Fig. 2A). No differences in the results and no heterogeneity were found on sensitivity analysis.

#### Intraoperative blood transfusion

Three studies encompassing 913 patients (228 who underwent MIPD and 685 who underwent OPD) compared intraoperative blood transfusion rates [15, 21, 23]. The pooled results showed a higher rate of intraoperative blood transfusion in the OPD group; the pooled RR (0.58; 95% CI 0.44 to 0.77; *P* = 0.0002) showed a significant difference in the intraoperative blood transfusion rate between the two groups. Heterogeneity was not significant (*I*^2^ = 0%) (Fig. 2B).

#### LOS

Six trials [15, 17, 20–23] with a total of 10,863 patients (1319 and 9544 who underwent MIPD and OPD, respectively) investigated the LOS. Five studies showed the LOS to be significantly lower in the MIPD group, whereas 1 showed it to be lower in the OPD group. The analysis found significant heterogeneity (*I*^2^ = 98%), and a random effects model was adopted. The pooled mean difference (MD = − 4.66; 95% CI − 9.13 to − 0.19; *P* = 0.04) indicated a significantly shorter LOS in the MIPD group (Fig. 2C). The results and heterogeneity were not significantly different in the sensitivity analysis.

#### Postoperative complications and 30-day mortality

Four studies that included 942 patients combined (243 who underwent MIPD and 699 who underwent OPD) [15, 17, 21, 23] examined surgical complications. The pooled surgical complication data revealed no difference between MIPD and OPD (RR = 0.84; 95% CI 0.67 to 1.05; *P* = 0.12, *I*^2^ = 45%) (Fig. 3A). A lack of significant differences was also observed on subgroup analyses of postoperative pancreatic fistulae (3 studies [15, 17, 21], RR = 0.91; 95% CI, 0.56 to 1.46; *P* = 0.69, *I*^2^ = 0%) (Fig. 3B), delayed gastric emptying (3 studies [15, 17, 21], RR = 0.86; 95% CI, 0.33 to 2.28; *P* = 0.77, *I*^2^ = 67%) (Fig. 3C), and postpancreatectomy hemorrhage (3 studies [15, 17, 21], RR = 1.33; 95% CI 0.68 to 2.58; *P* = 0.4, *I*^2^ = 0%) (Fig. 3D). Similarly, no significant difference in postoperative 30-day mortality was detected (RR = 1.01; 95% CI, 0.73 to 1.42; *P* = 0.93, *I*^2^ = 0%) in six studies [15, 17, 18, 20, 22, 23] that comprised 10,884 patients (1272 and 9612 who underwent MIPD and OPD, respectively) (Fig. 3E).

### Short-term oncological outcomes

#### Rate of vein resection

Three studies [15, 21, 23] comprising a total of 913 patients (228 underwent MIPD and 685 underwent OPD) provided data on vein resection; our analysis revealed no difference in the vein resection rate (RR = 0.96; 95% CI 0.76 to 1.20; *P* = 0.71, *I*^2^ = 0%) (Fig. 4A).

#### Rate of R0 resection

Eight studies [15–18, 20–23] including 11,192 patients (1349 and 9843 underwent MIPD and OPD, respectively) provided data regarding the R0 resection rate. We found that the R0 resection rate was higher in the MIPD group, with low heterogeneity as shown in a random effects model (RR = 1.06; 95% CI 1.01 to 1.12; *P* = 0.02, *I*^2^ = 36%) (Fig. 4B).

#### Harvesting and positivity rate of lymph nodes

The numbers of harvested lymph nodes were measured in 7 studies [15–18, 20, 21, 23] that included 9575 patients (1279 who underwent MIPD and 8296 who underwent OPD). There was no significant difference in the number of harvested lymph nodes between the two groups (MD 0.98; 95% CI − 1.32 to 3.27; *P* = 0.4) (Fig. 4C). The heterogeneity was high (*I*^2^ = 86%), and a random effects model was adopted. Moreover, data regarding lymph node positivity were also reported in these studies; the pooled results showed no statistical difference between the two groups (MD = 0.96; 95% CI 0.74–1.26; *P* = 0.78, *I*^2^ = 92%) (Fig. 4D). Our sensitivity analysis showed no obvious differences.

#### Time to starting adjuvant chemotherapy after surgery

Four studies [15, 20–22] gathered data on the time to starting adjuvant chemotherapy after surgery, including a total of 10,494 patients (1242 and 9252 underwent MIPD and OPD, respectively). The pooled results indicated that the time to adjuvant chemotherapy was significantly shorter in the MIPD group (MD = − 15.35; 95% CI − 30.33 to − 0.36; *P* = 0.04) (Fig. 4E). The heterogeneity was high (*I*^2^ = 98%), and a random effects model was adopted.

### OS and DFS

DFS data were available in 2 studies [15, 16]. A significantly longer DFS was observed in the MIPD group (HR 1.30, 95% CI 1.02 to 1.66, *P* = 0.04, *I*^2^ = 0%) (Fig. 5A). Eight studies [15, 16, 18–23] investigated OS; their pooled data revealed no significant difference between patients who underwent MIPD and those who underwent OPD (HR 0.98, 95% CI 0.95 to 1.02, *P* = 0.36, *I*^2^ = 0%) (Fig. 5B).

### Publication bias

**Fig. 6:**
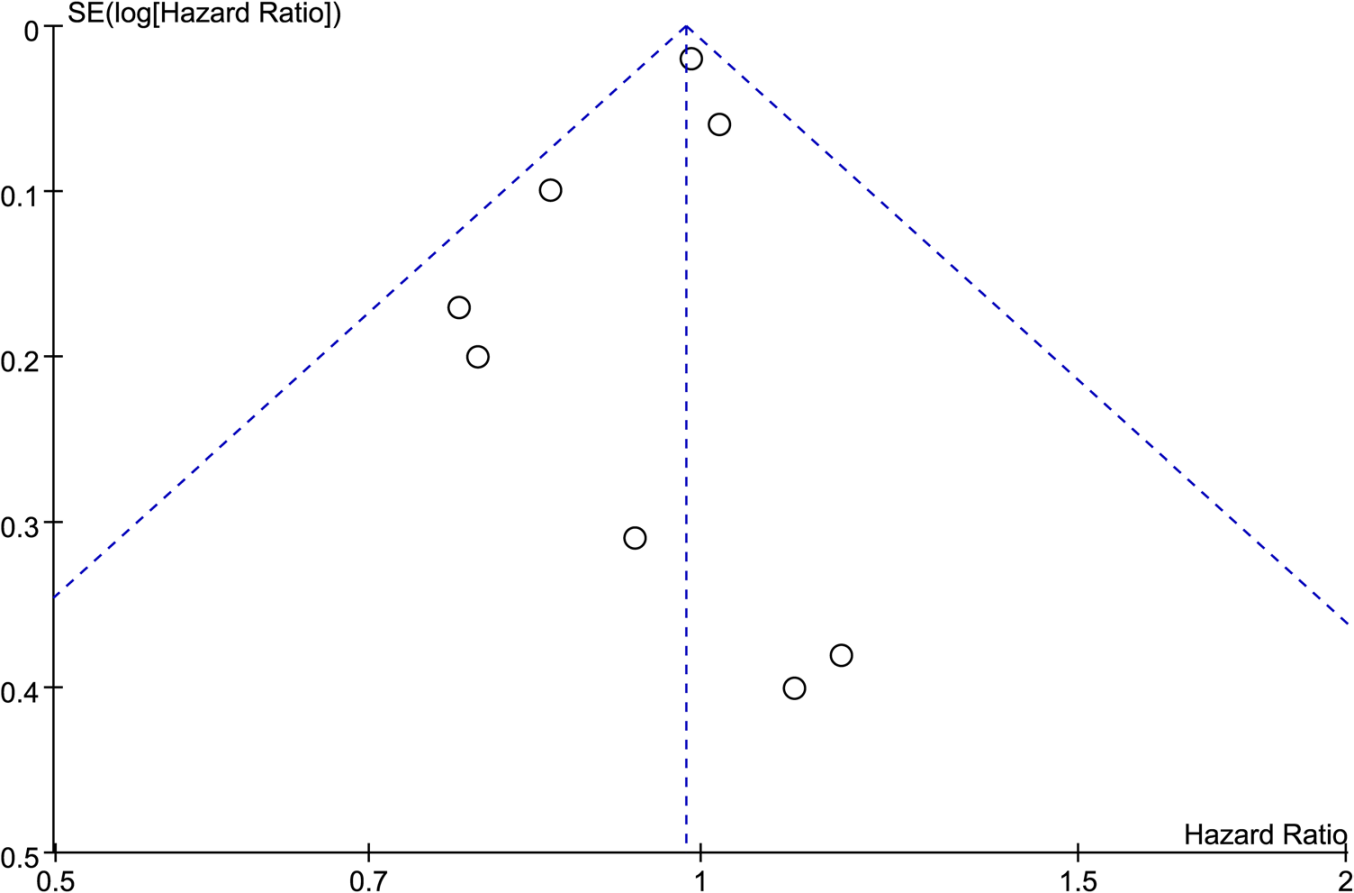
Begg’s funnel plot for assessing publication bias

Begg’s funnel plot was used to assess any publication bias present in the articles. As shown in the funnel plot of OS (Fig. 6), no evidence of significant publication bias was found.

## Discussion

To our knowledge, this is the first meta-analysis designed to specifically evaluate the perioperative and oncological outcomes of patients with PDAC who underwent MIPD and compare the findings with those for patients who underwent OPD. Overall, the pooled results revealed no significant difference in OS between the two groups, although patients who underwent MIPD had longer DFS periods and commenced adjuvant chemotherapy sooner after surgery. Moreover, we found no significant difference in operative time, postoperative complications, 30-day mortality, vein resection, number of harvested lymph nodes, or number of positive lymph nodes between the two approaches. There was a significant improvement in margin-negative resection with MIPD, as well as a shorter LOS and a lower intraoperative blood transfusion volume. Thus, our findings suggest that the outcomes of MIPD are at least equivalent to, if not better than, those of OPD in patients with PDAC.

It was previously thought that operating time would be longer with MIPD; this method is certainly challenging and has a steep learning curve. A recent retrospective multicenter analysis by Wang et al. [24] found that the learning curve for MIPD had 3 phases, with peaks evident after the completion of 40 and 104 cases. They also concluded that the operative time significantly decreased from phase I to phase III and was even comparable with that of OPD, consistent with the findings of van Hilst et al. [25] and Boone et al. [26]. In the present study, no significant difference between the 2 approaches was observed in terms of operative time. Croome et al. [15] excluded 10 patients who underwent MIPD in the first 6 months to avoid the early segment of their learning curve, which may have introduced bias with regard to our pooled results; however, we found no significant change in our results when excluding their study from our analysis. Therefore, our findings suggest that the operating time for MIPD is comparable to that of OPD once the learning curve is achieved.

The feasibility and safety of MIPD have been established given that enthusiasm for this procedure continues to grow quickly. MIPD has the potential to markedly reduce intraoperative blood loss and transfusion, as well as LOS; these attributes were corroborated in our own study. Although there were no sufficient data for analyzing intraoperative blood loss, the rate of blood transfusion was lower in patients undergoing MIPD in the present study, which ought to reflect lower intraoperative blood loss. Allogeneic blood transfusion has been confirmed to be an independent negative predictor of DFS and OS among patients with PDAC who underwent PD [27, 28]. Therefore, MIPD may potentially be related to improved survival compared to OPD in patients with this disease. A shorter LOS could also translate into faster recovery, which may help introduce subsequent treatments sooner. LOS is also an important component of healthcare costs [29], and whether opting for MIPD can significantly reduce such costs over OPD is an important area of active investigation [30].

In the present study, total and major complications attributed to MIPD occurred at a similar rate to those attributed to OPD, including postoperative pancreatic fistulae, delayed gastric emptying, postpancreatectomy hemorrhage, and 30-day mortality. Other studies have also observed such outcomes [25, 31, 32]. Therefore, the minimally invasive approach does not appear to alter the risk of severe complications and death compared to OPD. This is highly important because most resections are performed to treat malignant diseases. Moreover, Stauffer et al. showed that major complications may be an independent predictor of poorer survival [21]. It is highly probable that major postoperative complications would at least delay the administration of subsequent chemotherapy or limit the patient’s ability to tolerate a full course.

Our study also found that surrogate oncologic indicators including the number of resected veins, number of harvested lymph nodes, and rate of positive nodes were similar in the 2 groups, although the rate of R0 resection was higher in the MIPD group than in the OPD group. MIPD was contraindicated in patients who required vascular resection and reconstruction in a previous study [33]. However, Croome et al. [34] suggested that MIPD with major vascular resection is feasible and safe, and it can achieve similar results in terms of morbidity and mortality rates as well as of oncologic outcomes as does OPD with major vascular resection. The latter notion is supported by our data. Nevertheless, the acquisition of considerable experience for performing OPD both with and without vascular resection and for performing MIPD without vascular resection is strongly recommended before attempting minimally invasive major vein resection and reconstruction [35]. Previous studies found that the lymph node and margin statuses were significant predictors of DFS and OS [21, 36]. Our pooled results revealed that MIPD produced oncologic outcomes similar to, or even more favorable than, OPD did. Moreover, Stauffer et al. [21] found that the proportion of positive lymph nodes in patients who underwent MIPD was lower than that in patients who underwent OPD, and that the negative margin rate was higher; however, neither finding was statistically significant. These results may indicate that the minimally invasive approach allows for complete and adequate lymphadenectomy and pancreatic resection as it provides superior and magnified views of the tumor.

The primary results of this study showed a significant improvement in the time to starting adjuvant chemotherapy and in DFS among patients with PDAC who underwent MIPD; Conrad et al. [31] reported similar outcomes in their study. According to a recent evaluation of the European Study Group for Pancreatic Cancer, completion of all 6 cycles of adjuvant chemotherapy following resection of PDAC improves OS if the chemotherapy is started within 12 weeks [37]. Croome et al. [15] reported that the lack of chemotherapy within 90 days of surgery was a strong predictor of poorer OS; this suggests that the inability to initiate or complete chemotherapy in a timely manner will ultimately have a negative influence on survival. Our data do indicate a longer DFS in the MIPD group than in the OPD group, which may reflect the difference in the timing of adjuvant treatment initiation. Therefore, we posit that MIPD may enhance the ability of patients to receive subsequent treatments in a timely fashion after surgery and to complete their dosing schedules as well.

Another primary result in our study was the lack of a significant difference in OS between the 2 approaches. Chapman et al. [22] reported that patients undergoing MIPD had a noticeably longer median survival time than did those undergoing OPD (19.8 vs. 15.6 months) as well as an improved OS (HR 0.85, 95% CI 0.69 to 10.3) after adjusting for patient- and tumor-related characteristics; these findings were similar to those of Croome et al. [15] and Conrad et al. [31]. Furthermore, Stauffer et al. [21] also found that the 5-year survival rates of patients who underwent MIPD and OPD were 32% and 15.34%, respectively. However, the differences in OS in these aforementioned studies were not significant. It remains to be determined whether MIPD can result in a significantly different OS among patients with PDCA as additional patients are accrued and follow-up periods are lengthened. Nevertheless, in our opinion, one of the key objectives for achieving superior oncologic outcomes with existing treatment modalities is to improve the ability of patients to receive both complete tumor resection and chemotherapy, and we believe that MIPD is a realistic method to meet this objective.

Two other meta-analyses that were similar to ours were identified in our search [8, 38]. Although the stated aims of both were to assess LPD versus OPD in patients with PDAC, they included patients with ampullary carcinoma, some benign tumors, and even chronic pancreatitis in addition to those with PDACs. Moreover, the pooled results of short-term oncological outcomes such as R0 resection and number of lymph nodes retrieved were different from ours, since we only included patients with PDAC. Even though their findings regarding OS were similar to ours, they found 4 of the studies that were also included in our analysis [15, 20–22] to be heterogeneous (although we did not), whereas 8 other studies were not heterogeneous in their analyses (as consistent with our data). Consequently, their meta-analyses may prevent achieving objective conclusions regarding the oncologic safety of MIPD.

There were some limitations to our study that should be taken into account when considering the results. First, all included studies were retrospective and conducted in high-volume hospitals. Numerous studies have shown that hospital volume is significantly correlated with the incidence of perioperative outcomes [24]. Kantro et al. [20] reported that the 30-day mortality rate for patients who underwent MIPD was higher in low-volume hospitals than in high-volume institutions; therefore, there was a risk of selection bias even though such confounders could not be avoided. Second, the numbers of patients in some of the studies were too small, leading to low-power analyses. Third, only one of the included studies [16] compared RPD and OPD, which may have introduced bias because robotic and laparoscopic approaches have substantial differences, and the number of patients who underwent RPD was small and accounted for a small proportion in the analysis of results regarding the R0 resection rate, number of harvested lymph nodes, rate of positive nodes, and OS. However, the results and heterogeneity for all these variables showed no significant change after exclusion of the study comparing RPD and OPD. Nevertheless, some of our conclusions, particularly those pertaining to RPD, should be interpreted with caution. In the future, when more data are available, subgroup analysis should be performed to determine whether is any difference between the laparoscopic and robotic approaches. Finally, the heterogeneity in some of the results was high. Although we conducted sensitivity analyses, the results and heterogeneity were barely changed. Thus, some of our results should be interpreted with caution. Overall, additional prospective and multicenter randomized controlled trials with longer follow-up periods are warranted to compare the oncological outcomes of MIPD and OPD.

In conclusion, the results of our meta-analysis indicate that MIPD appears to be safe and feasible with perioperative, short-term, and long-term outcomes that are similar to those of OPD in the setting of PDAC. Moreover, MIPD may also provide advantages such as lower transfusion rates, higher rates of margin-negative resection, shorter time to starting adjuvant chemotherapy, and longer DFS when compared to OPD in patients with PDAC.

## Electronic supplementary material

Below is the link to the electronic supplementary material.

Supplementary file 1 (PDF 1898 kb)

Supplementary file 2 (DOCX 13 kb)

Supplementary file 3 (DOC 65 kb)
