# Supplementary material for: Perioperative and oncological outcomes following minimally invasive versus open pancreaticoduodenectomy for pancreatic duct adenocarcinoma
Source: Surg Endosc. 2020 Jul 6;35(5):2273–85. doi: 10.1007/s00464-020-07641-1 (PMC8057975; doi:10.1007/s00464-020-07641-1)
Supplement: Supplementary file 2 — (DOCX 13 kb) [file 464_2020_7641_MOESM2_ESM.docx]

**Supplementary2. Details of search strategy of Pubmed**

(((((((((((((Pancreaticoduodenectomies[Title/Abstract]) OR Pancreatoduodenectomy[Title/Abstract]) OR Pancreatoduodenectomies[Title/Abstract]) OR Duodenopancreatectomy[Title/Abstract]) OR Duodenopancreatectomies[Title/Abstract])) OR "Pancreaticoduodenectomy"[Mesh])) AND (((((((((((((((((((((Laparoscopies[Title/Abstract]) OR Celioscopy[Title/Abstract]) OR Celioscopies[Title/Abstract]) OR Peritoneoscopy[Title/Abstract]) OR Peritoneoscopies[Title/Abstract]) OR Surgical Procedures, Laparoscopic[Title/Abstract]) OR Laparoscopic Surgical Procedure[Title/Abstract]) OR Procedure, Laparoscopic Surgical[Title/Abstract]) OR Procedures, Laparoscopic Surgical[Title/Abstract]) OR Surgery, Laparoscopic[Title/Abstract]) OR Laparoscopic Surgical Procedures[Title/Abstract]) OR Laparoscopic Surgery[Title/Abstract]) OR Laparoscopic Surgeries[Title/Abstract]) OR Surgeries, Laparoscopic[Title/Abstract]) OR Laparoscopic Assisted Surgery[Title/Abstract]) OR Laparoscopic Assisted Surgeries[Title/Abstract]) OR Surgeries, Laparoscopic Assisted[Title/Abstract]) OR Surgery, Laparoscopic Assisted[Title/Abstract]) OR Surgical Procedure, Laparoscopic[Title/Abstract])) OR "Laparoscopy"[Mesh]))) OR (((((((((Procedure, Robotic Surgical[Title/Abstract]) OR Procedures, Robotic Surgical[Title/Abstract]) OR Robotic Surgical Procedure[Title/Abstract]) OR Surgical Procedure, Robotic[Title/Abstract]) OR Surgical Procedures, Robotic[Title/Abstract])) OR "Robotic Surgical Procedures"[Mesh])) AND (((((((Pancreaticoduodenectomies[Title/Abstract]) OR Pancreatoduodenectomy[Title/Abstract]) OR Pancreatoduodenectomies[Title/Abstract]) OR Duodenopancreatectomy[Title/Abstract]) OR Duodenopancreatectomies[Title/Abstract])) OR "Pancreaticoduodenectomy"[Mesh]))))) AND (("Carcinoma, Pancreatic Ductal"[Mesh]) OR (((((((((((((((((Carcinomas, Pancreatic Ductal[Title/Abstract]) OR Ductal Carcinoma, Pancreatic[Title/Abstract]) OR Ductal Carcinomas, Pancreatic[Title/Abstract]) OR Pancreatic Ductal Carcinomas[Title/Abstract]) OR Duct-Cell Carcinoma of the Pancreas[Title/Abstract]) OR Duct Cell Carcinoma of the Pancreas[Title/Abstract]) OR Pancreatic Ductal Carcinoma[Title/Abstract]) OR Pancreatic Ductal Carcinoma[Title/Abstract]) OR Pancreatic Duct Cell Carcinoma[Title/Abstract]) OR Carcinoma, Ductal, Pancreatic[Title/Abstract]) OR Duct-Cell Carcinoma, Pancreas[Title/Abstract]) OR Carcinoma, Pancreas Duct-Cell[Title/Abstract]) OR Carcinomas, Pancreas Duct-Cell[Title/Abstract]) OR Duct Cell Carcinoma, Pancreas[Title/Abstract]) OR Duct-Cell Carcinomas, Pancreas[Title/Abstract]) OR Pancreas Duct-Cell Carcinoma[Title/Abstract]) OR Pancreas Duct-Cell Carcinomas[Title/Abstract]))
